# The Cure of Leprosy

**Published:** 1905-04-29

**Authors:** 


### THE CURE OF LEPROSY.

Although, happily, leprosy is little more than a name to us in this country, it presents both in the characters of its causative organism and in the nature of its morbid processes such considerable analogies to tuberculosis, that the important researches of Captain E. R. Rost, I.M.S., are of more than academic interest even to us. Subject to confirmation by other observers, Captain Rost[1] seems to have satisfactorily achieved the first element of success in the investigation of microbic diseases by the discovery of a means of artificially cultivating the bacillus lepræ. This organism, which resembles the tubercle bacillus both in its resistance to decoloration and in its morphological characters, for long-defied cultivation. Captain Rost claims that the secret of its successful cultivation depends upon an absolute absence of chlorine from the medium. On such "a-chloretic" media he has been able to grow freely both the leprosy and tubercle bacillus. Following up his success he has made a "leprolin," a substance analogous to Koch's "old tuberculin," which contains the products of the bacillary metabolism, the organisms themselves being removed by filtration. This leprolin, when injected into a leper, causes a reaction in the leprous patches (which seems to remove all doubt as to the identity of the organism he has succeeded in growing), and appears to exercise a well-marked curative effect. With the failure of the old tuberculin as a curative agent fresh in our minds, we may well retain some scepticism as to the future of leprolin, but the reports to hand up to the end of last year certainly prompt the hope that a move has at last been made towards the successful treatment of one of the prime scourges of the East.

[1] Indian Med. Gaz., Dec. 1904.

